# Towards FLASH Radiotherapy in Lung Cancer: A Review on Preclinical Evidence and Technical Feasibility

**DOI:** 10.3390/cancers18152418

**Published:** 2026-07-27

**Authors:** Simone N. Visser, Steven J. M. Habraken, Anggraeini Puspitasari-Kokko, Anne H. zur Horst, Coen R. N. Rasch, Krista C. J. van Doorn-Wink

**Affiliations:** 1 Department of Radiation Oncology, Leiden University Medical Center (LUMC), 2333 ZA Leiden, The Netherlands; 2 Holland Proton Therapy Center (HollandPTC), 2629 JH Delft, The Netherlands; 3 Department of Radiation Oncology, Erasmus Medical Center (EMC), 3015 GD Rotterdam, The Netherlands

**Keywords:** lung cancer, FLASH radiotherapy, FLASH-effect, ultra-high dose rate, proton therapy, motion management

## Abstract

**Background/Objectives**: Alongside surgery and systemic treatments, radiotherapy plays a crucial role in the treatment of lung cancer but can affect healthy tissue and cause serious side effects. Compared to photon therapy, proton therapy can lower toxicity because protons deposit most of their energy within the tumor (the Bragg peak), sparing healthy tissue. FLASH radiotherapy (FLASH-RT), which is characterized by using ultra-high dose rates (UHDR), may further enhance healthy tissue sparing. Preclinical studies using electrons, protons, or photons have shown that FLASH-RT may reduce toxicity, while tumor response is maintained, known as the FLASH-effect. FLASH-RT with protons (FLASH-PT) is a promising treatment for lung cancer, integrating the biological advantages of FLASH with the physical advantages of proton beams. **Methods**: A literature search was performed on preclinical evidence of the FLASH-effect on lung-related endpoints, the proposed mechanisms, and treatment planning and delivery strategies for FLASH-PT in lung cancer. **Results**: Preclinical studies have shown reduced pulmonary fibrosis, inflammation, vascular damage, and oxidative stress with FLASH-RT. Current evidence suggests that the FLASH-effect likely arises from multiple radiochemical and biological processes, can vary between tissues, and depends on dose, beam and/or delivery characteristics. Despite growing evidence, the exact dose delivery conditions and technological factors required to obtain a FLASH-effect remain unclear. Treatment planning studies achieved clinically acceptable thoracic FLASH-PT plans for lung cancer, with similar or improved organ-at-risk sparing compared with conventional radiotherapy, while meeting (fraction) dose and dose rate criteria for the FLASH-effect. However, clinically feasible technologies for conformal dose delivery under UHDR conditions with respiratory motion mitigation are still under development. **Conclusions**: FLASH-PT holds the potential to reduce radiation-induced pulmonary side effects and expand the therapeutic window of radiotherapy. Still, key (pre)clinical and technological challenges must be addressed before clinical implementation for patients with lung cancer.

## Simple Summary

Lung cancer is often treated with radiotherapy, which can also damage healthy tissue and cause serious side effects. Proton therapy may reduce side effects in some patients by delivering radiation more precisely to the tumor. In recent years, FLASH radiotherapy (FLASH-RT) has gained substantial interest, as it may enhance healthy tissue sparing while maintaining tumor response through ultrafast dose delivery. Combining FLASH with proton therapy (FLASH-PT) is particularly promising for lung cancer. Preclinical studies demonstrated that FLASH-RT can reduce radiation side effects in healthy lung tissue. Although the exact mechanisms remain under investigation, multiple radiobiological processes are thought to contribute to this protective effect. Initial clinical studies have demonstrated that FLASH-PT appears technically feasible and may offer similar or better dose distributions than conventional radiotherapy techniques. However, important technological challenges remain, particularly related to dose conformity and handling breathing motion. Further research and technological developments are needed before clinical implementation.

## 1. Introduction

Radiotherapy is a common treatment for lung cancer, one of the most common and lethal malignancies [1]. However, radiation can also damage healthy tissue and cause serious side effects. In thoracic cancers, severe toxicities such as radiation pneumonitis (RP) and fibrosis could occur when healthy tissue surrounding the tumor is irradiated [2,3]. The therapeutic potential of radiotherapy is limited by the risk of radiation-induced (RI) toxicity.

Advances in technology and radiobiology have contributed to improvements in radiotherapy over the years [4]. This led to improved (image-guided) treatments, enhanced dose conformity, hypofractionation, adaptive radiotherapy, proton therapy, and more. With proton therapy (PT), radiation dose can be delivered more precisely to the tumor compared to more common conventional photon therapy. Protons deposit most of their energy at the end of their range, known as the Bragg peak (BP), and then come to a stop. Targeting the BP to the tumor maximizes tumor dose while fully sparing distal tissue and reducing integral dose, particularly relevant for large target volumes.

Over the past decade, FLASH radiotherapy (FLASH-RT) has gained considerable attention [5,6,7,8,9,10,11]. This technique involves delivering radiation at ultra-high dose rates (UHDR), typically exceeding 40 Gy/s, which is orders of magnitude larger than the dose rates used in conventional (CONV) radiotherapy. The dose rate threshold of 40 Gy/s originates from the early preclinical work in which the normal-tissue-sparing FLASH-effect was first observed, in particular, the marked reduction in lung fibrosis reported by Favaudon et al. at dose rates of ≥40 Gy/s compared with CONV dose rates [5]. It should be noted, however, that 40 Gy/s represents a widely adopted convention rather than a strict biological cut-off.

In preclinical studies, in a range of models, endpoints and irradiation modalities (electrons, photons and protons), FLASH-RT has shown reduced normal tissue toxicity while maintaining comparable tumor response [5,12,13,14,15]. This phenomenon, known as the FLASH-effect, has attracted significant interest for its potential to reduce RI toxicity and enhance the therapeutic bandwidth.

Combining FLASH with proton therapy (FLASH-PT), leveraging both their biological and physical advantages, holds promise as a novel treatment option. This narrative review focuses on lung cancer because it is a promising application for FLASH-PT. RI toxicity, including RP and pulmonary fibrosis, is dose-limiting in thoracic radiotherapy, making the normal-tissue-sparing effect of FLASH directly clinically relevant. At the same time, the lungs present challenges that FLASH-PT may help to address: respiratory motion complicates CONV PT through effects such as interplay [16], beam-tumor misalignment and the need for larger target volumes, while the high fraction doses used in stereotactic lung treatments [17] fall within the wide range of fraction doses considered suitable for FLASH-RT. Finally, substantial preclinical work has been conducted in lung-related endpoints, which will be discussed in this review. While preclinical studies were considered irrespective of irradiation modality, the technological scope of this review was restricted to proton therapy, as conventional electron beams cannot adequately treat deep-seated lung tumors and very high-energy electron (VHEE) and photon FLASH remain at an early stage of development.

While FLASH-RT research moves towards clinical implementation, it is crucial to recognize both the opportunities and the uncertainties [6]. This paper aims to provide a comprehensive overview of the current biological evidence and technological considerations related to FLASH-RT for lung cancer, identify the interconnections between these aspects, and highlight the remaining knowledge gaps.

## 2. Materials and Methods

This narrative review was conducted through a structured literature search in PubMed and Web of Science, using search terms on FLASH and UHDR combined with terms for lung cancer. Studies published from 2014 onwards were included, restricted to peer-reviewed original articles written in English. The search was last updated on 31 December 2025. The search strings are provided in Appendix A.

After removing duplicates, 214 articles were identified through database searching. Records were screened on title and abstract, after which full texts were assessed against the inclusion and exclusion criteria. Preclinical studies were included only if they investigated the effects of FLASH on lung cancer-related endpoints, regardless of the irradiation modality (electrons, photons, or protons). Preclinical studies were excluded when they did not investigate the FLASH-effect on a lung-related endpoint or used tumor cell types other than lung cancer cells. For the technological domain, only studies addressing the implementation and feasibility of cyclotron-based FLASH-PT in the context of lung cancer were included. Studies were excluded when they did not use UHDR, addressed treatment sites other than lung, or were not proton-based. All relevant publications were identified and categorized into either biological or technological topics. To identify further relevant publications, both backward and forward reference snowballing was performed. Following assessment of eligibility and relevance, 20 preclinical studies and 16 studies on treatment planning and delivery approaches were included.

## 3. Results

### 3.1. Preclinical Literature

In Table 1, the preclinical FLASH-RT studies with electrons, photons, or protons with lung (cancer) related endpoints, study parameters, and outcomes are listed.

#### 3.1.1. Preclinical Animal Studies Evaluating Pulmonary Effects of FLASH-RT

Although reduced radiation sensitivity at high dose rates was first reported over 60 years ago [37,38], interest in the field grew rapidly after Favaudon et al. (2014) demonstrated the FLASH-effect, as a differential effect between tumors and healthy tissue, in mice [5]. They showed a reduction in pulmonary fibrosis with FLASH-RT, while tumor growth repression of mouse lung carcinomas was equivalent to CONV-RT. In addition, FLASH-RT helped protect the blood vessels from apoptosis. Even after increasing the dose delivered in FLASH to 20 Gy, no detectable lung complications were noticed. Only when increasing the dose to 30 Gy did RI toxicities become evident. Using the same setup, Fouillade et al. (2020) observed reduced tissue injury, inflammation, and cellular senescence (i.e., a state of permanent cell cycle arrest in which cells remain metabolically active but can no longer divide) [18].

Gao et al. (2024) compared 100 Gy/s and 250 Gy/s dose rates in mice receiving whole-thorax irradiation of 20 or 30 Gy, delivered as a single fraction or split into two or four fractions separated by 8 min intervals [25]. With a total dose of 30 Gy delivered in a single fraction, the 250 Gy/s group showed reduced acute RP at 48 h and improved overall survival compared to the 100 Gy/s group. When the dose was split into two or four fractions, overall survival decreased significantly compared to the single-fraction delivery. A slight improvement was observed with four split fractions compared to two, though this difference was not significant. With a total dose of 20 Gy, no significant differences in survival or toxicity were observed between dose rates or split-dose schemes.

Dai et al. (2023) also examined the influence of split doses on the FLASH-effect in mice with xenografted lung tumors of A549 cells (human lung adenocarcinoma cell line) [24]. A single 20 Gy pulse or ten pulses of 2 Gy (with one minute in between pulses) showed similar tumor response under CONV and FLASH conditions, but both FLASH regimens resulted in significantly reduced RP at 72 h after irradiation. Since normal tissue sparing occurred with 2 Gy pulses, the authors concluded that the FLASH-effect can also be triggered at split doses of 2 Gy.

Extending these findings to immune-related endpoints, Tao et al. (2026) compared FLASH and CONV whole-thorax irradiation in mice using both single-fraction (17 Gy) and fractionated (2 Gy per day for 5 days) regimens [23]. Single-fraction FLASH irradiation showed higher lymphocyte counts, including T cells, NK cells, and B cells, and restored counts to near-baseline levels earlier than CONV. This immunoprotective advantage was maintained in the group receiving 2 Gy per fraction.

In the study of Ford et al. (2025), micro-CT revealed substantial reductions in lung function in healthy C57BL/6 mice after 30 Gy CONV (−50% functional residual capacity (FRC)), whereas 30 Gy FLASH caused smaller reductions (−30% FRC) [26]. Lung function was comparable between FLASH and CONV at a lower total dose of 15 Gy. Histology confirmed more extensive fibrosis in the 30 Gy CONV group than in the 30 Gy FLASH group, whereas no significant changes were observed at 15 Gy. More recently, Lu et al. (2025) irradiated C57BL/6 mice to 17.8 Gy whole-thorax irradiation with electrons under FLASH or CONV conditions [22]. They reported that mice irradiated with FLASH showed better preservation of pulmonary function than those irradiated with CONV. Furthermore, their results showed less acute (1 day and 7 days after irradiation) and less long-term (1, 3, and 6 months after irradiation) lung injury with FLASH.

Lee et al. (2026) irradiated healthy C57BL/6 mice with a single proton spot and a high fraction dose of 60 Gy under FLASH and CONV conditions [29]. They demonstrated that FLASH reduced fibrosis progression and maintained lower levels of oxidative stress and inflammatory markers compared to CONV throughout the 90-day observation period. However, there was no significant difference in overall survival.

Finally, a known side effect of thoracic radiotherapy is RI heart disease, associated with a decrease in overall survival [39]. Furthermore, heart dose has been identified as a predictive factor for RP [40,41]. Kim et al. (2024) studied cardiac effects in healthy C57BL/6 mice exposed to 40 Gy CONV or FLASH [28]. They showed that cardiac function was preserved better and fibrosis was reduced with FLASH at 8 and 30 weeks after irradiation.

#### 3.1.2. Preclinical Studies Using Human Lung Tissue

Fouillade et al. also studied human lung fibroblasts (MRC5 and IMR90) and epithelial carcinoma cells (A549) exposed to 5.2 Gy FLASH electrons [18]. They observed significantly reduced DNA damage markers (53BP1) and lower cell lethality in the FLASH group at 30 min after irradiation. Furthermore, primary bronchial epithelial cells (PBEC) were irradiated at 2 or 4 Gy using FLASH or CONV. The PBEC irradiated with FLASH showed significantly less radiation-induced differentiation and cell death at the 4 Gy dose point at 21 days after irradiation.

In the study of Buonanno et al. (2019), normal human lung fibroblasts (IMR90) were irradiated with different doses and dose rates (0.05, 100, and 1000 Gy/s) [33]. Overall cell survival did not significantly differ for the increased dose rates. However, at the highest dose (20 Gy), FLASH-RT significantly reduced the formation of γH2AX foci at 30 min after irradiation. Moreover, inflammatory markers were reduced for the highest dose rate at 24 h after irradiation, and the number of senescent cells was reduced for both FLASH groups one month after irradiation compared to CONV.

Adrian et al. (2021) investigated normal lung fibroblasts (MRC5) exposed to doses ranging from 3 to 12 Gy with FLASH and CONV dose rates under normoxic conditions [30]. Their findings showed a better surviving fraction for FLASH, but this difference was not significant.

Guo et al. (2022) investigated normal lung fibroblasts (IMR90) and lung cancer cells (A549) exposed to 15 Gy with FLASH and CONV [34]. They found higher cell viability in normal fibroblasts after FLASH compared to CONV at 7 days after irradiation, while lung cancer cells showed lower viability after FLASH. Furthermore, the indicator for oxidative stress showed increased reactive oxygen species (ROS) levels after CONV irradiation, but no significant change was noticed after FLASH.

Del Debbio et al. (2025) studied the FLASH-effect in human PBEC from healthy lungs and tumor cells [31]. Healthy cells showed a higher surviving fraction when irradiated with FLASH compared to CONV. The same analysis was performed on A549 tumor cells, and showed no significant variation in survival fraction, indicating that the tumor cell response after FLASH and CONV was similar. FLASH reduced markers associated with RI pulmonary fibrosis (e.g., vimentin), suggesting less normal tissue damage with FLASH.

In the ex vivo study of Dubail et al. (2025), lung tissue samples from lobectomy patients were irradiated with CONV or FLASH [32]. FLASH preserved more proliferating cells (EdU+) at 24 h after irradiation in 95% of the samples. Moreover, FLASH limited the effect on cell cycle arrest and showed less activation of oxidative stress pathways, suggesting reduced ROS generation and better normal tissue preservation.

Kuipers et al. (2025) evaluated FLASH-PT in COPD patient-derived PBEC organoids [35]. No differences between FLASH and CONV were observed for most endpoints; however, organoids irradiated with FLASH exhibited slightly worse organoid-forming capacity, which suggests less tissue regenerative potential with FLASH. The authors mentioned the absence of immune cells, endothelial cells, and fibroblasts in the model, and the use of COPD-derived cells, which may respond differently to radiation than healthy cells, as possible reasons for this unexpected finding.

Velalopoulou et al. (2025) conducted an ex vivo pilot study with FLASH-PT on normal human lung tissue from three healthy donors [36]. The precision-cut samples were irradiated with a single fraction of 12 Gy with FLASH and CONV. Their results showed metabolic differences between the modalities and between the two male and one female donor. However, both findings need to be validated in a larger cohort.

#### 3.1.3. Effect on Lung Tumor Microenvironment

Kim et al. (2021) studied the differential effects of FLASH and CONV irradiation on the tumor microenvironment in mice bearing subcutaneously implanted Lewis lung carcinoma (LLC) tumors [19]. CONV irradiation induced vascular collapse within the tumor, which is associated with tumor hypoxia and may limit treatment efficacy. FLASH irradiation, however, did not cause vascular collapse, suggesting better preservation of the tumor vasculature. Interestingly, FLASH produced higher levels of ROS than CONV in the tumor, which is considered beneficial as ROS can damage tumor cells and promote cell death. Despite higher ROS levels, FLASH-irradiated tumors showed reduced γH2AX foci at 6 h after irradiation, indicating differences in DNA damage dynamics. FLASH was associated with increased T-cell infiltration into the tumor, suggesting a more effective immune response compared to CONV. The exact mechanisms underlying these differential effects between FLASH and CONV remain unclear, though the role of myosin light chain (MLC) signaling, associated with tumor progression [42], was further investigated by the authors. CONV irradiation activated MLC, while FLASH did not. When MLC activation was blocked during CONV irradiation, the results were similar to those seen with FLASH. This suggests that MLC activation plays a key role in the differential effects observed in the tumor between FLASH and CONV irradiation.

Shukla et al. (2023) examined tumor response with a single FLASH and CONV dose of 18 Gy in mice injected with LLC cells [27]. Mice irradiated with FLASH showed a greater reduction in tumor cell numbers at 5 days and 8 days after irradiation, less proliferation (fewer Ki67-positive cells at day 8), higher apoptosis, and more DNA damage markers (γH2AX) at both time points. Additionally, with FLASH, more immune cells were attracted into the tumor, with an increased ratio of immune to tumor cells and higher T-cell infiltration. These results suggest that FLASH outperformed CONV in suppressing tumor growth while boosting the antitumor immune response. However, another study contradicts a major role of the immune response in the antitumor efficacy of FLASH: Almeida et al. compared immune responses between FLASH-RT and CONV in orthotopic lung tumors in immunocompetent and immunodeficient mice [21]. In this study, tumor growth was delayed similarly for both modalities across immunocompetent and immunodeficient mice. Furthermore, a similar radiation-induced remodeling of the tumor microenvironment was observed (e.g., similar lymphoid and myeloid cell distributions, no increase in intratumoral transforming growth factor-β1 levels (TGF-β1)).

### 3.2. Insights into the Mechanisms of the FLASH-Effect in Lung Tissue

Preclinical studies investigated both the presence and magnitude of the FLASH-effect across various endpoints and treatment conditions, as well as the underlying biological mechanisms responsible for normal tissue sparing. It is desirable to better understand what is causing the FLASH-effect in lung cancer before its clinical implementation [7]. Although multiple hypotheses have been proposed, current evidence suggests that no single mechanism fully accounts for the FLASH-effects [43,44].

#### 3.2.1. Oxygen Depletion

The oxygen depletion hypothesis is one of the most studied explanations for the FLASH-effect. It proposes that the ultra-fast energy deposition of UHDR causes rapid oxygen depletion within tissues, reducing the availability of oxygen for ROS formation and thereby limiting indirect DNA damage from ROS production [45]. This is closely linked to the concept of oxygen enhancement, describing how oxygen amplifies RI DNA damage [46]. By transiently depleting oxygen, UHDR may effectively reduce the radiosensitivity of normal tissue, while tumor tissues, often already hypoxic, derive less benefit. FLASH delivery has been associated with lower levels of ROS production in some studies [29,31,32]. However, the oxygen depletion theory alone cannot fully account for the FLASH-effect. Fraction doses as low as 2 Gy were sufficient to induce a FLASH-effect in mice, a scenario that theoretically should not produce substantial oxygen depletion in tissue with relatively high oxygen tension, such as normal lung tissue [24,47,48]. Leavitt et al. (2024) reported that FLASH demonstrated greater effectiveness against hypoxic lung tumors in mice compared with CONV [20]. These findings underline that additional biological processes must contribute to the observed healthy tissue sparing with FLASH.

#### 3.2.2. Mitochondrial Protection

Mitochondria are a major intercellular source of ROS and play central roles in apoptosis, metabolism, and cellular stress responses. Consequently, limiting mitochondrial damage may reduce oxidative stress and attenuate downstream cell death pathways following irradiation. Supporting this hypothesis, several studies showed reduced DNA damage markers with FLASH compared with CONV [18,33,34,49]. Guo et al. showed that human lung fibroblasts irradiated in vitro with CONV exhibited greater mitochondrial damage than those exposed to FLASH-PT [34]. Preservation of mitochondria may therefore contribute to reduced oxidative stress and limit cell death pathway activation and promote cellular recovery after irradiation and thus contribute to the normal tissue sparing observed with FLASH-RT.

#### 3.2.3. Immunomodulation

Another aspect is the involvement of the immune response, representing an additional category of biological effects that potentially contributes to both normal tissue protection and tumor control. With single-cell RNA sequencing of more than 130,000 cells, including myeloid, lymphoid, mesenchymal, epithelial, and endothelial cells, Lu et al. identified broad immunological differences between CONV and FLASH in the lungs of mice at one week after irradiation [22]. This finding indicates that FLASH influences multiple cellular compartments involved in tissue homeostasis and repair.

Studies in tumor models have reported that FLASH-RT can enhance antitumor immunity characterized by increased immune cell infiltration, while also showing a decrease in immunosuppressive cells [27]. This could support tumor control by promoting immune-mediated tumor cell elimination. However, these findings were not consistent across all studies, as others have reported similar RI immune remodeling following CONV or FLASH irradiation [21]. Therefore, the precise role of immune modulation in normal tissue sparing and tumor control is not yet clearly defined.

#### 3.2.4. Cellular Senescence

Accumulating evidence suggests that FLASH-RT may influence cellular senescence, an important biological process associated with RI tissue injury. For example, FLASH-RT demonstrated a differential effect on inflammatory responses and protected healthy cells from RI senescence [27]. This preservation of cellular function may help maintain the regenerative capacity of lung tissue, while reducing the pro-inflammatory signaling that contributes to the development of fibrosis [28,29,44].

Overall, the findings suggest that the FLASH-effect cannot be explained by a single biological mechanism. Oxygen depletion, mitochondrial protection, immune modulation, and reduced cellular senescence are likely to interact to produce a FLASH-effect. The relative contribution of each mechanism probably depends on irradiation parameters, tissue type, and biological context. Consequently, further characterization of the FLASH-effect—defining the physical and biological conditions at which it occurs and the magnitude of the effect across different biological models—remains an essential step towards clinical translation [50]. Notably, data suggest that the FLASH-effect can vary between tissues [51,52], indicating that the underlying biological mechanisms are likely tissue-specific and remain incompletely understood [44,53].

**Figure 1:**
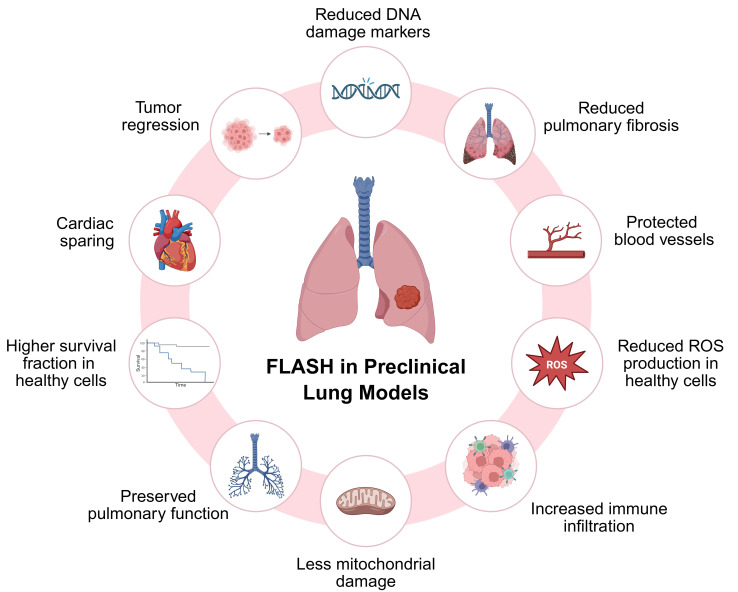
Effects in lung tissue-related endpoints seen in preclinical studies with FLASH radiotherapy.

All observed effects in the preclinical studies are summarized in Figure 1.

### 3.3. Treatment Delivery Approaches

#### 3.3.1. UHDR-PT as Modality

Radiotherapy involves trade-offs between target coverage and sparing organs-at-risk (OAR), and UHDR introduces a new dimension: achieving high dose rates, required for FLASH, compromises dose conformity. For lung cancer, sufficient penetration depth to reach the tumor adds yet another constraint. Among the available modalities, FLASH-PT offers a promising balance between these requirements [54]. As synchrotron-based proton systems operate under different delivery constraints and are less commonly used for FLASH research to date, this study focuses on cyclotron-based systems. Of the included studies on treatment planning and delivery approaches, the majority investigated pencil beam scanning systems. With current cyclotron-based proton therapy, UHDR can only be achieved at the highest energy of 230–250 MeV. As a result, the beam shoots through the patient, instead of stopping inside the target, known as transmission beams (TB). Using TBs causes dose distal to the target, but the sharp lateral penumbra can be advantageous in certain cases, and TB delivery is insensitive to range uncertainty arising from tissue heterogeneity, which is particularly relevant in the thorax [55]. Clinical feasibility and safety of UHDR with TB (UHDR-TB) have been tested in the FAST-01 and FAST-02 trials on bone metastases in the extremities and in the thorax [56,57], respectively.

**Figure 2:**
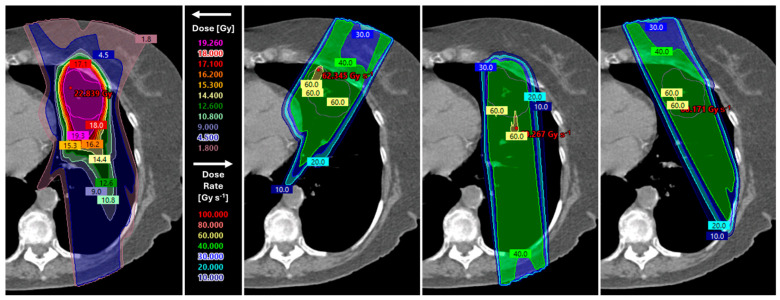
Dose distribution (**left**) and corresponding dose rate distribution (**right**) for a FLASH Bragg-peak treatment plan using 3D range modulators and a collimator for one fraction of 18 Gy (in the context of a 3 × 18 Gy stereotactic body radiotherapy (SBRT) treatment for lung).

Using UHDR with Bragg peaks (UHDR-BP) limits dose beyond the target through the characteristic distal dose fall-off but is more sensitive to range uncertainties than UHDR-TB. A further challenge is that BPs require multiple energies, whereas current energy-switching times are too slow to maintain UHDR [58]. Therefore, the high-energy beams need to be adjusted with static range modulators, range shifters, and apertures creating UHDR-BPs, which is under active development and approaching clinical implementation [59]. In Figure 2, an example of a dose and dose rate distribution is shown.

In preclinical studies, irradiation parameters are often not directly translatable to clinical practice, e.g., dose rates used in preclinical settings sometimes exceed what can currently be achieved with clinical PT set-ups. Therefore, alternative dose rate metrics have been proposed for clinical UHDR-PT. However, distinctions exist between the dose rate definitions. The pencil beam scanning dose rate (PBS-DR) takes into account the spatiotemporal delivery of the dose [60], whereas the average dose rate (ADR) accounts for the entire treatment field, including the time between individual spot deliveries, resulting in a considerably lower effective dose rate. Since each voxel typically receives dose from multiple sequentially delivered spots, the dose-averaged dose rate (DADR) offers an approach to accumulate and weight dose rate contributions in each voxel with the corresponding dose [61]. Other commonly used dose rate metrics include the dose threshold dose rate (DTDR), which only evaluates the dose rate for dose contributions exceeding a dose threshold, and the spot peak dose rate (SPDR), which uses the maximum instantaneous dose rate delivered during an individual spot. In Table A1 in Appendix B, the dose rate metrics for all included studies are listed.

PBS-DR can be increased/maximized by using delivery pattern optimization (DPO). Substantial effort has been devoted to optimizing spot delivery sequences to achieve UHDR conditions with current technology [62,63,64]. In the study of José Santo et al. (2023), TB with DPO can achieve PBS-DR up to 80 Gy/sec at the outer edges of lung tumors of varying volume [62]. UHDR is desired at the tumor boundary because it may trigger the FLASH-effect in the surrounding healthy tissue. With DPO, the local dose rate increased, and with it, the FLASH-coverage (volume receiving > 40 Gy/s (V_40Gy/s_)) reached 29%, compared with 6.9% with a standard pattern. In this study, a nozzle proton current of 40 nA was used, while higher nozzle currents can yield even higher dose rates.

Diao et al. (2025) studied the treatment parameters for UHDR-BP [65]. In a phantom study, they found that the impact of DPO was influenced by the number of beams and the beam arrangement. The 3-field beam arrangement showed better optimization effects. The ADR in the esophagus, heart, spinal cord, and lungs-GTV was significantly improved by DPO.

These technological developments showed that UHDR-PT can be delivered with existing proton machines and optimized to achieve high dose rates, making it a standalone modality with clinical potential, even before considering the biological benefits of the FLASH-effect.

#### 3.3.2. FLASH-Compatible UHDR-PT Planning and Delivery

In radiotherapy, the dose is usually delivered in multiple fractions over several weeks. Advances in treatment techniques enabled better protection of healthy tissue, allowing higher fraction doses and fewer fractions, known as hypofractionation.

In lung cancer, this includes approaches like stereotactic body radiotherapy (SBRT) [17]. Since the FLASH-effect appears to be reduced by fractionation [50,66], high fraction doses are preferred. The FLASH-effect typically requires a FLASH threshold of 3.5–7 Gy per fraction [67]. While the sparing effect increases with dose [52], evidence also suggests it reaches a saturation point at high doses [5,68].

Habraken et al. (2022) assessed the feasibility of stereotactic UHDR-PT with a single-beam-per-fraction (SBPF) [69]. Different fraction doses and numbers of beams were used. They showed that beam doses above the FLASH threshold can be delivered at FLASH-compatible dose rates. They found a break-even FLASH enhancement ratio (FER) of approximately 1.3 for the equivalent dose in 2 Gy fractions (EQD_2_) to healthy lung tissue and a FER of 1.1–1.3 for the conformity of EQD_2_. Based on reported lung fibrosis data in mice (FER > 1.8) [5], the study suggested that a FLASH-effect is achievable in stereotactic UHDR-PT of lung lesions.

Continuing the idea of Habraken, the study of Zeng et al. (2024) used 11 Gy uniform fraction dose (UFD) TBs with SBPF, but also optimized for non-UFD [70]. The dose per beam, i.e., the fraction dose, varied for the non-UFD plans from 5.0 to 24.2 Gy. One beam delivered most of the dose, and the other four beams delivered less dose. Non-UFD outperformed UFD by combining conventional fractionation effects with enhanced FLASH-effects, achieving more OAR sparing without compromising target coverage. Later, the same research group did a similar study with UHDR-BP plans instead of UHDR-TB [71]. They compared UHDR-BP with UFD of 10.0 Gy and non-UFD ranging from 5.0 to 20.0 Gy. In both types of plans, a high dose conformity was found, and non-UFD showed better OAR sparing than UFD. Overall, optimizing fraction dose while using an SBPF could contribute to OAR sparing by using both the benefits of fractionation and reaching the requirements for the FLASH-effect.

#### 3.3.3. UHDR/FLASH Treatment Planning Evaluation in Lung Cancer

Treatment planning studies aim to assess the performance of UHDR-TB and/or UHDR-BP, compared to each other or to clinically used treatment planning techniques. Clinically used techniques combine intensity modulation with angular degrees of freedom, enabling highly conformal dose distributions, while sparing surrounding healthy tissue. Volumetric modulated arc therapy (VMAT) and intensity modulated proton therapy (IMPT) are common current-state-of-the-art techniques for photons and protons, respectively. While FLASH-RT may improve the conformity of biologically effective FLASH-enhanced dose, it is still unacceptable to exceed dose constraints for critical OARs. Comparing planning between VMAT, IMPT, and current and near-future UHDR-compatible techniques provides insights into the dosimetric trade-offs and the range of possible clinical applications of the latter. In Table A1 in Appendix B, the studies comparing UHDR treatment planning to conventional planning and delivery approaches in lung cases are listed.

Van Marlen et al. (2020) evaluated stereotactic UHDR-TB for seven lung cancer patients [72]. They reported similar or better sparing in the lungs, thoracic wall, and heart compared to VMAT, while having similar target coverage. Schwarz et al. (2022) compared UHDR-TB to IMPT for one lung cancer case [54]. Similar dose to the target and the OARs were observed, and the high dose conformity was improved with UHDR-TB. However, the integral dose was 33% higher with UHDR-TB compared to IMPT.

In a follow-up study, Van Marlen et al. (2022) performed a planning study comparing VMAT, IMPT, and UHDR-TB [73]. The UHDR-TB provided similar or improved dose distributions compared to VMAT, but was inferior to IMPT, showing better sparing for the ribs, skin, and low-dose lung volumes (volume receiving < 10 Gy).

Wei et al. (2022) evaluated the robustness of UHDR-TB with respect to both dose and ADR [74]. Plans with three beams showed robust target coverage comparable to or slightly worse than IMPT, while robustness improved with five beams and became comparable to IMPT. Dose rate robustness was high for both groups, with ±5 mm setup and ±3.5% range uncertainties, causing less than 3% variation in the V_40Gy/s_ for healthy lung tissue.

The group of Wei et al. (2022) also compared UHDR-BP with IMPT [58]. Single-energy UHDR-BP could achieve dose distributions comparable to IMPT, which relies on multi-energy BP beams. Less dose conformity was observed for UHDR-BP compared to IMPT. However, no significant differences were observed for any of the evaluated OARs. Furthermore, the V_40Gy/s_ was evaluated across dose thresholds of 0.1, 1, and 5 Gy. At all thresholds, the highest V_40Gy/s_ among the evaluated OARs was consistently achieved by the lung-GTV. As the dose threshold increased, the V_40Gy/s_ increased too, ultimately reaching 100% for all OARs—including the esophagus, heart, and spinal cord—at the highest dose threshold of 5 Gy. Wei et al. (2021) performed a comparison between UHDR-BP and UHDR-TB [75]. The dose rate was comparable between the two techniques, but UHDR-BP showed better OAR sparing. A similar study was done by the group of Kang et al. (2022), comparing UHDR-TB and UHDR-BP [76]. They showed less uniformity with UHDR-BP, but improved dose-volume metrics for the lungs, spinal cord, heart, and esophagus compared to UHDR-TB.

The study of van Marlen et al. (2024) evaluated three UHDR-BP approaches: five beam plans with (1) a single energy layer with pristine BP pencil beams, (2) generic ridge filter, and (3) 3D range modulators [77]. UHDR-BP plans were compared with UHDR-TB and IMPT for different target sizes. Although high-quality and high-dose-rate plans could be achieved with all UHDR-BP approaches, none demonstrated a clear overall advantage. UHDR-TB showed a lower mean lung dose and a higher percentage of volume delivered in the FLASH thresholds (dose rate > 40 Gy/s and dose > 4 Gy). The UHDR-BP approaches did reduce integral dose and dose to other OARs compared to UHDR-TB but remained inferior to IMPT. Only minor differences were observed between the UHDR-BP techniques, with the 3D range modulator approach showing slightly better results.

Ma et al. (2023) did a feasibility study on hybrid FLASH, where BP and TB are combined [78]. Hybrid plans significantly reduced the dose to the lungs and the spinal cord compared to TBs only. All plan types had a V_40Gy/s_ of 100% in the target, where only TBs also achieved a V_40Gy/s_ = 100% in all OARs, which was >85% for the hybrid plans.

Finally, simultaneous dose and dose rate optimization has been investigated by Gao et al. for UHDR-TB [79,80]. To create FLASH-compatible treatment plans, hypofractionation, i.e., high fraction dose to achieve UHDR, and multiple beams were required to achieve dose distributions similar to IMPT. Studies have similarly shown that a higher dose is associated with a higher FLASH-coverage [75,79]. However, Kang et al. (2021) reported that a lower fraction dose (15 Gy instead of 34 Gy) resulted in higher V_40Gy/s_ in the target volume [81].

#### 3.3.4. Breathing Motion

Patient breathing causes lung tumor motion during treatment. For large breathing amplitudes, mitigation strategies are required because conventional delivery typically takes several minutes and spans tens of breathing cycles. Also, breathing motion may lead to under- or overdosing through the interplay effect [82]. Internal margins can be applied to expand the clinical target volume (CTV) into an internal target volume (ITV) that typically encompasses all tumor positions as delineated on the phases of a 4D planning CT, or treatment can be planned on the phase best representing the average tumor position, known as the mid-ventilation, with adequate additional margins to account for residual motion uncertainty [83].

UHDR treatments, however, involve sub-second delivery times (see Appendix B, Table A1), shorter than an average breathing cycle of 3–4 s [84]. As a result, the amplitude of motion during irradiation is reduced, and the interplay effect is partially mitigated. This provides the possibility of planning directly on the CTV with only a small residual motion margin rather than the ITV, reducing dose to healthy tissue and associated side effects. This makes UHDR-PT promising for moving targets such as lung tumors, on top of the biological advantages of the FLASH-effect [85].

To reduce margins safely, the tumor position during irradiation needs to be known, either through direct imaging of the target volume or a surrogate external signal with a known correlation to the internal anatomy. One approach is respiratory gating, where radiation is delivered only within a predefined gating window [86,87]. When the tumor moves outside this window, the beam is paused, allowing smaller margins and less dose to surrounding OARs [88]. In conventional radiotherapy, this prolongs treatment time due to repeated beam-on/beam-off, but this is less relevant with the very short treatment times of UHDR-PT. Breathing motion is often irregular and can be improved with assisted-breathing solutions, such as active breathing control (ABC), which makes the breathing more reproducible and can improve gating accuracy [89]. With sufficient guidance, combining UHDR-PT with gating is a promising motion management strategy [90].

An alternative approach is breath-hold (BH), where patients either voluntarily or with assistance hold their breath in inspiration or expiration to suppress tumor motion. Given the short delivery times of UHDR-PT, BH is particularly attractive, as the required BH duration is much shorter than in conventional PT. Van Marlen et al. suggested in their study that dose-volume outcomes could have been improved when combined with a short BH [73]. However, voluntary BH performance varies between patients and depends on patient compliance, necessitating imaging or motion monitoring to ensure accuracy [91]. While relatively short BHs during UHDR treatment should be feasible for lung cancer patients, pre-treatment imaging remains challenging, as (cone-beam) CT acquisition typically takes several minutes. This can be circumvented by acquiring images over several BHs with pauses in between [92,93,94], or by using a prolonged BH facilitated by supplemental oxygen [95]. With oxygen support, the BH can last up to 5 min [96], potentially enabling both tumor-verification imaging and UHDR treatment within a single BH. However, increased oxygen levels may influence the magnitude of the FLASH-effect and should be carefully considered [97].

An additional approach is tumor tracking, where the tumor position is determined in real time, and the beam adapts accordingly [98]. This can be done with or without implanted fiducial markers [99], although marker-less approaches are preferred due to the risk of a pneumothorax. However, marker-less tracking requires advanced imaging and motion prediction, which may limit clinical implementation. Given the short delivery times of UHDR-PT, the remaining motion is expected to be small, potentially limiting the added value of complex tracking.

**Figure 3:**
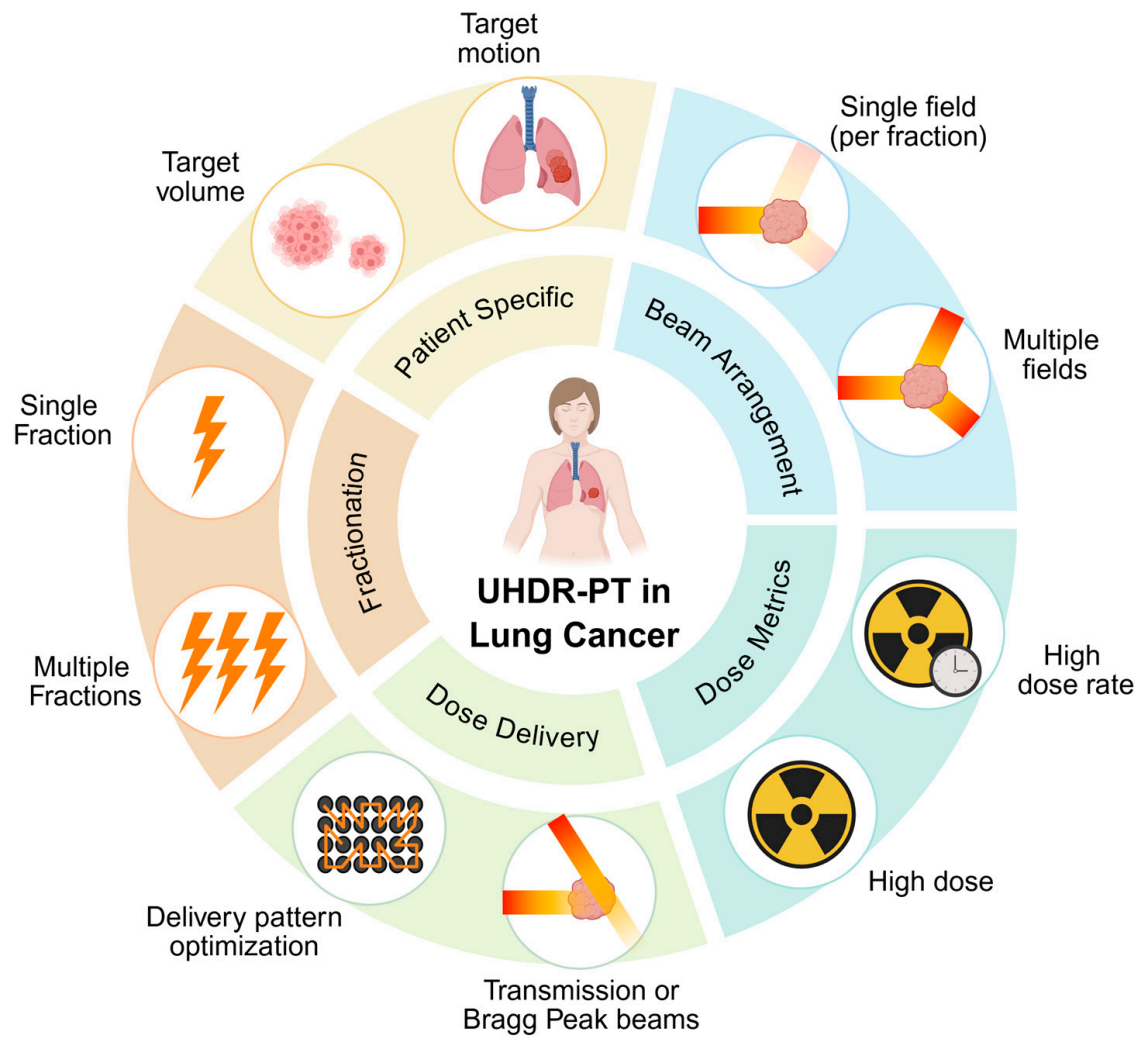
Overview of treatment parameters considered for ultra-high dose rate proton therapy in lung cancer.

In Figure 3, the treatment parameters considered for UHDR-PT for lung cancer, and as discussed in this section, are summarized.

## 4. Discussion

Across electrons, photons, and protons, preclinical in vivo studies have demonstrated that FLASH-RT has the potential to reduce normal lung tissue toxicity, while preserving lung tumor response, thereby widening the therapeutic window. Beneficial effects have been observed across multiple endpoints, including pulmonary fibrosis, inflammation, vascular injury, oxidative stress, functional impairment, and immune preservation. Lung tumor response was generally equivalent between FLASH and CONV dose rates and even improved with FLASH-RT in one study [27]. The FLASH-effect was observed across all three modalities. The studies by Kristensen et al. (2025) and Almeida et al. (2024) show no difference between electron FLASH and FLASH-PT in mouse skin and brain, respectively [100,101], suggesting that the results obtained with electrons can likely be translated to protons. In vitro and ex vivo studies using human lung cells and tissue further support the normal-tissue-sparing potential of FLASH-RT. Reduced DNA damage, oxidative stress, senescence, and fibrosis-related responses have been observed in healthy lung cells and tissues, while tumor cell responses generally remained comparable to CONV irradiation. Although results vary between models, differences in tissue origin and underlying comorbidities may contribute to the observed variability. Nevertheless, these studies provide important mechanistic and translational support for the clinical development of FLASH-RT. Still, multiple factors continue to pose challenges to the clinical implementation of FLASH-RT for lung cancer. For FLASH-PT in general, dose and dose rate requirements and the use of multiple fields and fractionations appear to be the most crucial knowledge gaps [102], which is consistent with remaining uncertainties requiring further research specifically for FLASH-PT in lung cancer.

### 4.1. Dose and Dose Rate Thresholds

A key uncertainty in translating FLASH to clinical lung cancer treatment is which combination of dose and dose rate is required to trigger the FLASH-effect. Both parameters are used to define the FLASH-effect, yet neither has been established as a discrete threshold, and the two cannot be considered independently.

The evidence is, however, inconsistent in both dimensions. Some studies observed no differential effect for fraction doses ≤ 20 Gy but a significant effect at 30 Gy [25,26], while others report effects at fraction doses as low as 2 Gy, a dose level more commonly used in PT [23]. Similarly, some studies reported no difference in survival with increased dose rates [33], whereas others do observe a dose rate-dependent effect [25]. Furthermore, the extremely high dose rates in studies that have reported the FLASH-effect are sometimes higher (exceeding 2000 Gy/s) than what can be achieved with clinical proton machines for large volumes (up to 100 s Gy/s). This difference may limit the magnitude of FLASH sparing that can be realized clinically.

Several methodological factors may account for this apparent disagreement. Most importantly, dose rates are not defined uniformly across studies. The definitions used are outlined in Section 3.3, and since they are not directly comparable, the accurate interpretation of dose rate parameters across studies requires attention and standardization. In addition, the pulse structure of the beam and how the beams or segments are delivered differ between irradiation modalities and treatment planning and delivery approaches. As a result, the same (average) dose rate can correspond to very spatiotemporal delivery dynamics. Together, this not only complicates the translation of preclinical FLASH dose rate thresholds to clinical PBS delivery but also hinders direct comparison between studies that use different dose rate metrics. Kang et al. (2021) compared three dose rate metrics (DADR, ADR, and DTDR) for two hypofractionation regimens (54 Gy/3 fx and 34 Gy/1 fx) and their effect on FLASH-coverage [81]. DADR yielded 100% FLASH-coverage in both the tumor and OARs for both fraction doses. ADR and DTDR showed lower FLASH-coverage; interestingly, lowering the fraction dose increased FLASH-coverage with these metrics, which the authors explained by the reduced number of spots required to deliver 15 Gy compared to 34 Gy. For both ADR and DTDR, V_40Gy/s_ could be improved by increasing the spot weight, reported as MU/spot, as a higher spot weight directly raises the instantaneous dose rate (167 Gy/s and 670 Gy/s for the low and high beam weight conditions, respectively). Notably, it remains unclear which dose rate definition is most relevant for predicting the FLASH-effect.

Taken together, the current evidence supports a dependency of the FLASH-effect on both dose and dose rate but does not identify a threshold for either. Yet, these parameters are required for predicting the FLASH-coverage in a treatment plan. The optimal combination of fraction dose and dose rate for lung (tumor) tissue therefore remains to be established.

### 4.2. (Hypo)fractionation

When interpreting preclinical evidence, a distinction must be made between studies observing a FLASH-effect delivered in multiple pulses or split doses [24,25], and those truly fractionated with sufficient time between fractions [23], as the biological response to dose delivered in pulses may not be equivalent to fractionated delivery.

Based on the available (preclinical) data, some form of hypofractionation is likely required to maximize the benefits of FLASH. However, especially in locally advanced settings, conventionally fractionated regimens are still the standard of care. A high dose per fraction can lead to serious toxicity in patients with centrally located tumors if the FLASH sparing effect is not reproduced clinically.

Quantifying the interaction between fractionation and sparing furthermore requires a radiobiological model. The classical linear-quadratic (LQ) model does not account for dose rate effects above conventional dose rates and therefore cannot describe FLASH without modification. Böhlen et al. addressed this using the biologically effective dose (BED), deriving the break-even sparing that the FLASH-effect must provide for a hypofractionated UHDR schedule to outperform a CONV fractionated schedule of equivalent tumor response [103]. Habraken et al. applied a comparable approach at the treatment plan level for lung cancer, expressing the FLASH-effect as a FER applied to the fraction dose in all OAR voxels [69]. As SBPF delivery enables UHDR but increases the effective fraction dose, they derived the break-even FER at which the FLASH-effect compensates for this loss. Rather than predicting the benefit of FLASH-PT for lung cancer, these frameworks make explicit what the FLASH-effect must achieve, which is what lung-specific FMFs will have to be tested against.

### 4.3. Biological Variability and the Limits of the Available Models

Whether the sparing observed preclinically will be reproduced in patients depends on biological variability that is not yet completely understood. In animals, the genetic background can determine whether the FLASH-effect occurs at all. Mice lacking telomerase (*Terc*^−/−^), which have very short telomeres, showed no sparing of lung fibrosis, while wild-type mice irradiated with the same setup were clearly spared [18]. These mice were genetically modified rather than naturally different, but the finding shows that these factors can remove the effect completely.

Evidence in humans is far more limited. No clinical data from lung cancer patients treated with FLASH are available. The available human evidence is limited to in vitro and ex vivo models, with nearly all such studies reporting differential effects between FLASH and CONV. However, some variability has been observed. For example, one study reported differences in metabolic responses among donor lungs from two male and one female donor [35], although the sample size was far too small to support any meaningful conclusions. In another study, organoid-forming capacity was slightly worse after FLASH [35]. Notably, these PBEC organoids were derived from COPD patients, raising the question of whether pre-existing lung disease influences the FLASH-effect.

The underlying limitation is that studies with human cells or tissue, while more directly relevant than animal models, cannot capture the physiology of a living organism. Advanced human lung tissue models, such as alveolus-on-a-chip systems, offer valuable opportunities to further investigate the tissue-sparing effects of FLASH-RT and the underlying biological mechanisms and dependencies that drive them. By providing insights that are more directly relevant to human physiology, these models can support the safe and effective clinical translation of FLASH for lung cancer treatment. They can also complement animal studies, which have significantly advanced—and continue to advance—our understanding of FLASH-RT, while addressing the limitations associated with extrapolating findings from animal models to human biology [104,105].

### 4.4. Combination with Systemic Therapy

Systemic therapy is standard of care for many patients with lung cancer. However, the interaction between FLASH-RT and (concurrent) systemic therapy (e.g., chemotherapy, immunotherapy, targeted agents) in lung tissue remains largely unknown. This represents one of the largest gaps between the current preclinical evidence and clinical practice. Ideally, systemic therapy would enhance tumor control, while preserving the normal-tissue-sparing effects of FLASH-RT. Combined treatment strategies may be particularly promising in combination with immunotherapy. Because FLASH-RT delivers the radiation dose within milliseconds, it may reduce radiation-induced lymphopenia, thereby better preserving immune function and potentially enhancing the efficacy of systemic therapy [106]. Supporting this concept, a melanoma model demonstrated that combining FLASH-RT with immunotherapy reduced tumor growth and improved survival, without increasing treatment-related toxicity [107]. Nevertheless, future research is needed to determine whether these benefits translate to the lung and to characterize the pulmonary effects of FLASH when combined with systemic therapies.

### 4.5. Achieving UHDR in Clinically Realistic Lung Plans

Even if the biological requirements were fully known, delivering them within clinical constraints remains a separate challenge, involving trade-offs between delivery approach, target volume, and beam arrangement.

The available evidence indicates that UHDR-PT is feasible, either with TB or by implementing additional hardware and creating BPs. First treatment planning studies showed similar or improved dose distribution with UHDR-TB and UHDR-BP compared with VMAT. UHDR-BP reduces the dose distal to the target and lowers the integral dose compared with UHDR-TB, but may come at the expense of dose conformity [58]. However, various included studies have shown that UHDR-BP achieved sufficient conformity under optimized conditions. Still, generating BPs at UHDR requires patient- and beam-specific range modulators, which increases technological complexity and may complicate clinical implementation. In comparison, UHDR-TB is technically less complex to implement in clinical practice and is more robust to range uncertainties, at the cost of a higher integral dose. In both delivery methods, triggering the FLASH-effect is influenced by biological and physical parameters such as fractionation, delivery pattern, and beam arrangements. Ultimately, all parameters and considerations for achieving UHDR and triggering the FLASH-effect, while maintaining dose conformity, should be integrated into treatment planning optimizers [79,80]. However, this requires a (more) precise definition of the FLASH-effect and the working mechanisms, especially in human cells or tissues, highlighting the intersection of radiobiology and physics in FLASH research.

### 4.6. Motion Management Under UHDR Conditions

An additional benefit of UHDR-PT is the potential to reduce the impact of respiratory motion, which remains an important consideration in radiotherapy for lung cancer. Accurate irradiation of a moving lung tumor with a FLASH proton beam in milliseconds offers an opportunity to freeze intrafraction motion, reduce treatment volumes, and (partly) mitigate the interplay effect, but at the same time poses a technical challenge. Residual tumor motion still needs to be considered during UHDR delivery. For this reason, (controlled) respiratory gating will be essential. Other strategies, such as BH, could provide additional means of motion management.

The benefit of UHDR on motion mitigation is largely reasoned rather than demonstrated. Treatment planning studies quantifying the effect of UHDR combined with motion mitigation on the dose distribution are therefore needed, alongside simulation studies of motion mitigation strategies and their accuracy under UHDR conditions, such as the gating accuracy achievable at UHDR.

### 4.7. Future Perspective

Future research in FLASH-RT for lung cancer should focus on further translating preclinical findings into clinical implementation. As outlined in the Introduction, several features make lung cancer a suitable candidate for FLASH-PT. This is supported by the Delphi consensus study from Klaver et al. on clinical studies with FLASH [108]. This tumor site aligns well with the consensus criteria, since the benefits of FLASH have been demonstrated in lung-related endpoints in preclinical studies, high fraction doses are already commonly applied in clinical practice, UHDR delivery may offer advantages for motion management, and treatment can be delivered using TBs, BPs, or a hybrid combination. Furthermore, lung cancer is associated with significant early and late toxicities that are detectable on imaging, facilitating the assessment of the FLASH-effect. However, treatment planning studies to date have mainly focused on SBRT lung cases, which generally involve small tumor volumes for which achieving UHDR is feasible. Larger tumor volumes, as typically seen in PT, may pose a limiting factor for achieving UHDR [58,73].

Besides protons, other modalities for UHDR treatment are under investigation, e.g., VHEE FLASH [109], spatially fractionated proton therapy (SFPT) FLASH [110], and FLASH with carbon ions [111], which might lead to new insight regarding FLASH-RT for lung cancer.

## 5. Conclusions

Preclinical data from multiple institutions indicate that FLASH-RT provides a significant sparing effect on healthy pulmonary tissue compared with conventional radiotherapy. The next step is to translate these findings into clinical practice for patients with lung cancer by enrolling them in well-designed clinical trials supported by a strong radiobiological rationale. Regarding delivery parameters, preclinical evidence suggests a dose, dose rate, and fractionation dependency of the FLASH-effect. However, the evidence from preclinical work is still too limited to fully rely on the FLASH-effect to compensate for the loss in, e.g., dose conformity, suggesting that conformal plans at least equivalent to IMPT should be a prerequisite for FLASH-PT. The treatment planning studies showed very promising results for UHDR-TB, UHDR-BP, or hybrid UHDR approaches to make FLASH-PT for lung cancer possible in the near future.

Further research should focus on the exact requirements for the FLASH-effect in scenarios closer to the clinical setting, both on the radiobiology side—such as models that more closely reflect human tissue and the combination with systemic treatments—and on the treatment delivery side—such as larger and moving tumors.

## Figures and Tables

**Table 1:** Preclinical studies investigating FLASH irradiation with lung-related endpoints. (PBEC, pulmonary bronchial epithelial cells; LLC, Lewis lung carcinoma; RP, radiation pneumonitis).

Author, Year	Model	Type of Particle	Energy (MeV)	Total Dose		Dose Rate		Fractionation	FLASH Sparing	Endpoint
				CONV (Gy)	FLASH (Gy)	CONV (Gy/s)	FLASH (Gy/s)			
Favaudon, 2014 [5]	C57BL/6J mice and TC-1 murine lung tumor model	Electrons	4.5	15 or 17	17	0.03	60	1 × 17 Gy	Yes	Reduced fibrosis and apoptosis in normal tissue; equal tumor growth inhibition
Fouillade, 2020 [18]	C57BL/6J and *Terc*^−/−^ mice	Electrons	4.5	17	17	0.03	60	1 × 17 Gy	Yes	Reduced tissue damage, inflammation, and senescence
Kim, 2021 [19]	Tumor-bearing C57BL/6 mice (LLC)	Electrons	16	15	15	0.06	352	1 × 15 Gy	-	Differential effect in tumor microenvironment between FLASH and CONV
Leavitt, 2024 [20]	Tumor bearing C57BL/6J and Swiss nude mice (SV2)	Electrons	6	20	20	0.1	100	1 × 20 Gy	-	FLASH more effective against hypoxic tumors
Almeida, 2024 [21]	Tumor bearing C57BL/6J, Swiss nude and NRG mice (SV2(-OVA) and H454)	Electrons	5.5	20	20	0.1	≥2000	1 × 20 Gy, 2 × 6 Gy or 3 × 8 Gy	-	Tumor response largely dose rate independent; immune response not a major contributor to FLASH antitumor efficacy
Lu, 2025 [22]	Healthy C57BL/6 mice	Electrons	6	17.8	17.8	0.3	200	1 × 17.8 Gy	Yes	Reduced acute and long-term lung injury with FLASH, less inflammation, boosted immune response, and enhanced tissue regeneration
Tao, 2026 [23]	Healthy C57BL/6 mice	Electrons	9	17	17	0.3	340	1 × 17 Gy	Yes	Higher lymphocyte counts and faster recovery (nearly one month) with FLASH
	Healthy C57BL/6 mice	Electrons	9	10	10	0.3	1.4 × 10^6^	5 × 2 Gy	Yes	Higher lymphocyte counts and faster recovery (one week) with FLASH
Dai, 2023 [24]	Tumor bearing Balb/c mice (A549)	Photons	1–2	20	20	0.03	200	1 × 20 Gy (one pulse) or 10 × 2 Gy (10 pulses)	Yes	Less severe RP with FLASH; similar pulmonary pathology in both FLASH groups
Gao, 2024 [25]	Healthy Balb/c mice	Photons	8	-	20	-	100 or 250	1 × 20 Gy; 2 × 10 Gy or 4 × 5 Gy	No	No differences in survival or toxicity
	Healthy Balb/c mice	Photons	8	-	30	-	100 or 250	1 × 30 Gy; 2 × 15 Gy or 4 × 7.5 Gy	Yes	Reduced RP with 250 Gy/s and better survival when delivered in 1 fraction
Ford, 2025 [26]	Healthy C57BL/6 mice	Photons	10	15	15	0.04	90	1 × 15 Gy	Yes	Similar lung function; no significant differences in tissue damage
	Healthy C57BL/6 mice	Photons	10	30	30	0.06	98	1 × 30 Gy	Yes	FLASH better preserved lung function and less extensive fibrosis
Shukla, 2023 [27]	Tumor-bearing C57BL/6 mice (LLC)	Protons	244 (CONV) 250 (FLASH)	18	18	1	60	1 × 18 Gy	Yes	FLASH reduced proliferation; increased DNA damage, cell death, and immune cells in the tumor
Kim, 2024 [28]	Healthy C57BL/6 mice	Protons	230	40	40	0.8	120	1 × 40 Gy	Yes	FLASH preserved cardiac function; reduced progression of fibrosis
Lee, 2026 [29]	Healthy C57BL/6 mice	Protons	230	60	60	2	500	1 × 60 Gy	Yes	Lower levels of oxidative stress and inflammatory markers; reduced pulmonary fibrosis; reduced skin toxicity
Fouillade, 2020 [18]	Normal human lung fibroblasts (MRC5 and IMR90) and lung cancer cells (A549)	Electrons	4.5	5.2 ± 0.2	5.2 ± 0.2	0.03	60	1 × 5 Gy	Yes	Reduced DNA damage markers in normal cells with FLASH
	Human PBEC from lobectomy patients	Electrons	4.5	2 or 4	2 or 4	0.03	60	1 × 2 Gy or 1 × 4 Gy	Yes	Preserved PBEC with FLASH
Adrian, 2021 [30]	Normal human lung fibroblasts (MRC-5)	Electrons	10	3–12	3–12	0.23	>800	1 × 3 Gy; 2 × 3 Gy; 3 × 3 Gy or 4 × 3 Gy	No	No difference in survival fraction
Del Debbio, 2025 [31]	Human PBEC (16HBE) and lung cancer cells (A549)	Electrons	8	2 or 4	2 or 4	0.1	275	1 × 2 Gy or 1 × 4 Gy	Yes	Reduction in fibrosis markers, cell death, cell cycle arrest and ROS levels with FLASH
Dubail, 2025 [32]	Normal human lung tissue from lobectomy patients	Electrons	7	9	9	0.5	450	1 × 9 Gy	Yes	Preserved cell proliferation; reduced DNA damage, cell cycle arrest, and oxidative stress with FLASH
Buonanno, 2019 [33]	Normal human lung fibroblasts (IMR90)	Protons	4.5	1, 2, 5, 10 or 20	1, 2, 5, 10 or 20	0.05 or 0.2	100 or 1000	1 × 1 Gy; 1 × 2 Gy; 1 × 5 Gy; 1 × 10 Gy or 1 × 20 Gy	Yes	FLASH reduced DNA damage markers, cell senescence and inflammatory markers; no difference in survival or acute effects
Guo, 2022 [34]	Normal human lung fibroblasts (IMR90) and lung cancer cells (A549)	Protons	4.5	15	15	0.33	100	1 × 15 Gy	Yes	FLASH better preserved normal cells; FLASH improved tumor cell kill; prevented mitochondrial damage
Kuipers, 2025 [35]	COPD-derived PBEC organoids	Protons	250	2 or 8	2 or 8	0.25	40	1 × 2 Gy or 1 × 8 Gy	No	No difference in DNA damage; slightly worse organoid-forming capacity with FLASH
Velalopoulou, 2025 [36]	Normal human lung tissue from donors	Protons	230	12	12	0.6	95	1 × 12 Gy	-	Metabolic differences in early response between FLASH and CONV

## Data Availability

No new data were created or analyzed in this study. Data sharing is not applicable to this article.

## Abbreviations

The following abbreviations are used in this manuscript:

ABC	Active breathing control
ADR	Averaged dose rate
BH	Breath-hold
BP	Bragg peak
CONV	Conventional
COPD	Chronic obstructive pulmonary disease
CT	Computed tomography
CTV	Clinical target volume
DADR	Dose-averaged dose rate
DPO	Delivery pattern optimization
DTDR	Dose threshold dose rate
EQD_2_	Equivalent dose of 2 Gy fractions
FER	FLASH enhancement ratio
FLASH-PT	FLASH proton therapy
FLASH-RT	FLASH radiotherapy
FRC	Functional residual capacity
GTV	Gross target volume
Gy	Gray (SI unit)
IMPT	Intensity modulated proton therapy
ITV	Internal target volume
LLC	Lewic lung carcinoma
MeV	Mega electron volt
MLC	Myosin light chain
ms	Millisecond
MU	Monitor units
nA	Nanoamperes
OAR	Organs-at-risk
PBEC	Primary bronchial epithelial cells
PBS-DR	Pencil beam scanning dose rate
PT	Proton therapy
RI	Radiation induced
ROS	Reactive oxygen species
RP	Radiation pneumonitis
SBPF	Single-beam-per-fraction
SBRT	Stereotactic body radiation therapy
SFPT	Spatially fractionated proton therapy
SPDR	Spot peak dose rate
TB	Transmission beams
(non)UFD	(non)uniform field dose
UHDR	Ultra-high dose rate
UHDR-BP	Ultra-high dose rate with Bragg peak
UHDR-PT	Ultra-high dose rate proton therapy
UHDR-TB	Ultra-high dose rate with transmission beams
VHEE	Very high-energy electrons
VMAT	Volumetric modulated arc therapy

## Appendix A

A search was conducted in PubMed and Web of Science. The following limits were applied to all databases: English language, peer-reviewed journal articles only, publication date from 2014 onwards.

### Appendix A.1. PubMed Search String

(FLASH[tiab] OR FLASHRT[tiab] OR “Ultra high dose rate*”[tiab] OR UHDR[tiab]) AND (“Lung Neoplasms”[Mesh] OR SCLC[tiab] OR NSCLC[tiab] OR ((“Lung”[Mesh] OR lung[tiab] OR lungs[tiab] OR pulmonar*[tiab]) AND (neoplasms[mesh] OR cancer*[tiab] OR carcinom*[tiab] OR malignan*[tiab] OR maligne[tiab] OR malignes[tiab] OR metasta*[tiab] OR neoplas*[tiab] OR oncolog*[tiab] OR tumor[tiab] OR tumors[tiab] OR tumour*[tiab]))).

### Appendix A.2. Web of Science Search String

(FLASH OR FLASHRT OR “Ultra high dose rate*” OR UHDR) AND (SCLC OR NSCLC OR ((“Lung” OR lung OR lungs OR pulmonar*) AND (cancer* OR carcinom* OR malignan* OR maligne OR malignes OR metasta* OR neoplas* OR oncolog* OR tumor OR tumors OR tumour*))).

## Appendix B

Table A1 provides an overview of FLASH treatment planning studies in lung cancer.

**Table A1:** Treatment planning studies on lung cancer cases with different delivery techniques, fractionation regimens, and other parameters. Parameters with ‘-’ indicate that this is not specifically mentioned in the study. Abbreviations: BP, Bragg peak; TB, transmission beam; SBFT, single-beam-per-fraction; ms, milliseconds; SPDR, spot peak dose rate; DADR, dose-averaged dose rate; ADR, average DR; DTDR, dose threshold dose rate; PBS-DR, pencil beam scanning dose rate; TADR, time-averaged dose rate; SESOBP, single energy spread-out Bragg peak.

Author, Year	Delivery Technique	Total Dose (Gy)	Fraction Dose (Gy)	Beam Dose (Gy)	Dose Rate (Gy/s)	Dose Rate Metric	Number of Beams	Fractionation	Energy (MeV)	Delivery Time (ms)	Target Volume (cm^3^)	Number of Cases
Van Marlen, 2020 [72]	TB	54	18	-	≥40	SPDR	10	3	244	300–730	Average 15.4 (range: 4.6–34.6)	7
Gao, 2020 [80]	TB	2	2	-	≥40	DADR	1, 3	1	229	-	-	3
	TB	6	6	-	≥40		3, 5, 9	1	229	-	-	3
	TB	10	10	-	≥40		5, 9, 17	1	229	-	-	3
Wei, 2021 [75]	TB	34	34	-	≥40	ADR, DADR and DTDR	5	1	250	-	Median: 54.5 (range: 22.8–194)	10
	TB	54	18	-	≥40		4	3	250	-		10
	BP	34	34	-	≥40		5	1	250	-		10
	BP	54	18	-	≥40		4	3	250	-		10
Kang, 2021 [81]	TB	34	34	-	≥40	ADR, DADR and DTDR	5	1	240	<1000	Median: 61 (range: 24–226)	9
	TB	45	15	-	≥40		5	3	240	<1000		9
Habraken, 2022 [69]	TB	54	18	18	141	Mean field dose rate (equivalent to ADR)	3	3—SBPF	244	127	Median: 6.4 (range: 4.4–10.1)	12
	TB	65.5	13.1	13.1	132		5	5—SBPF	244	99		12
	TB	73.7	10.5	10.5	136		7	7—SBPF	244	77		12
	TB	90	8.9	8.9	133		9	9—SBPF	244	67		12
Gao, 2022 [79]	TB	36	12		≥40	DADR	3	3	229	-	-	1
Van Marlen, 2022 [73]	TB	34	34	-	≥40 or ≥100	DADR, PBS-DR and ADR	5	1	250	60–310	1.39–8.58	6
										160–1470	54.54	1
Wei, 2022 [74]	TB	34	34	-	>40	ADR	3, 5	1	250	-	Median: 86.7 (range: 24.4–194.4)	10
Wei, 2022 [58]	BP	34	34	-	>40	ADR	3	1	250	-	Median: 54.5 (range: 22.8–194)	10
Schwarz, 2022 [54]	TB	60	20	-	>40	DADR and sliding window	3	3	230	-	31	1
Kang, 2022 [76]	TB	34	34	-	>40	SPDR	1, 2, 3 or 5	1	250	-	-	6
	BP	34	34	-	>40		4	1	250	-	-	6
Ma, 2023 [78]	TB	34	34	-	>40	DADR	3	1	250	1365	85 ± 43	5
	TB + BP	34	34	-	>40		3	1	250	1366		5
	TB + SESOBP	34	34	-	>40		3	1	250	1360		5
José Santo, 2023 [62]	TB	54	18	18	>40	PBS-DR	3	3–SBPF	244	622–3876	Median: 8.7 (range: 4.4–84)	20
Zeng, 2024 [70]	TB	55	11	11	148.5	TADR	5	5—SBPF	236	85.5	Average: 15.4 (range: 3.5–33.4)	11
	TB	55	11	5–24.2	123.3		5	5—SBPF	236	79.5		11
Van Marlen, 2024 [77]	TB	34	34	-	>40	PBS-DR and sliding window	≤5	1	250	-	2.1, 9.5 and 50.5	1
	BP *	34	34	-	>40		≤5	1	250	-		1
Zeng, 2025 [71]	BP	50	10	10	>40	PBS-DR	5	5—SBPF	150–218	359	-	15
	BP	50	5–20	5–20	>40		5	5—SBPF	150–218	332	-	15

* BPs consisted of three different orders of complexity: pristine BPs, generic ridge filter, and 3D range modulator.
